# Physiology

**Published:** 1878-08

**Authors:** 


### Summary.

*Collaborators:*

Dr. H. Gradle, Dr. L. W. Case, Dr. R. Park,
Dr. R. Tilley, Dr. D. R. Brower.

### Physiology.

Action of Heat and Cold on the Organism.—P. Delmas. (*Bull. de l'Acad. de Médecine, No.* 22.)

The author communicated to the *Académie de Médecine*, session of May 28th, a paper on this subject, of which the following is a résumé:

1st. Pending the administration of a cold douche, preceded or not by the use of heat, made at the temperature of 10° C. to 25° C. and of a duration from 30 seconds to five minutes, the central temperature of the body and that of the intermediary zone *are not at all or but very little lowered.*

2nd. While rest during the hours after administration of cold water does not facilitate the pretended reaction admitted by authors, and although the subject feels only a very moderate degree of heat, or of coolness, and sometimes even chills, the temperature of the central and intermediate parts lowers very little, or rises and passes that felt before the douche; the rapidity of the heart increases and the arterial tension remains very high.

3rd. Exercise following a cold douche has the true physiological result of bringing on persistent reduction of temperature and even a diminished rapidity of pulse and lowering of arterial tension.

4th. Under the influence of a cold application, the maximum and minimum of rapidity of the heart correspond to the maximum and minimum of arterial tension.

5th. After the application of a cold douche, the subject in reality cools, and his temperature lowers while he feels a sensation of heat, or, on the contrary, it rises or remains stationary when he has chills.

Physiology of the Pancreas.—Affanassien and Pawlon. (*Pflüger's Archiv.*—Note in *Lancet.*)

The authors reasoned by analogy that this gland received special excitory fibers as well as a vaso-motor system of nerves. The existence of the latter is indubitable; that of the former they proved thus: control experiments made by testing the juice just after feeding and during fasting, showed that the secretion of fluid and solid constituents is due to two different processes, governed by two sets of nerves, by one of which the chemical work is regulated. Resorting to Heidenhain's plan, they injected atropine, and the secretion was either checked or inhibited. They consider this positive evidence of the existence of secretory nerves. They next proceeded to enquire whether, admitting the accuracy of Bernstein's statement that irritation of the vagus inhibited the pancreatic secretion, this inhibitory influence is due to direct action, or is referable to the pain caused by stimulation of the nerve. Their experiments satisfied them that sensory irritation of the skin was capable of inhibiting the secretion of the pancreas.

